# Proportion and Associated Factors of Low Vision among Adult Patients Attending at University of Gondar Tertiary Eye Care and Training Center, Gondar Town, Ethiopia

**DOI:** 10.1155/2020/7042905

**Published:** 2020-05-28

**Authors:** Melkamu Temeselew Tegegn, Gizachew Tilahun Belete, Ayanaw Tsega Ferede, Aragaw Kegne Assaye

**Affiliations:** University of Gondar, College of Medicine and Health Science, Department of Optometry, P.O. Box 196, Gondar, Ethiopia

## Abstract

**Introduction:**

Low vision is a worldwide health problem in both developing and developed countries. A national survey of low vision and blindness in Ethiopia showed that the prevalence of low vision was 3.7% and that of blindness was 1.6%, whereas there is no evidence in the study area.

**Purpose:**

The study was aimed to assess the proportion and associated factors of low vision at the University of Gondar tertiary eye care and training center.

**Methods:**

A hospital-based cross-sectional study was conducted on 727 study participants with a systematic random sampling technique from April 18 to May 16, 2019. Data were collected through the use of a structural questionnaire and physical eye examination. Data were entered into Epi Info version 7, and analysis was performed by using statistical package for social science (SPSS) version 20. The binary logistic regression model was fitted to identify factors associated with low vision, and variables with a *P* value of <0.05 in a multivariable binary logistic regression were considered as statistically significant.

**Results:**

A total of 715 study participants have participated in this study with a mean age of 49.39 ± 19.93 years. The prevalence of low vision was 35.7% (95% CI: 32.3, 39.3). Being female (AOR = 1.58; 95% CI: 1.10, 2.28), no formal educational level (AOR = 2.24; 95% CI: 1.25, 4.02), history of cataract surgery (AOR = 2.58; 95% CI: 1.53, 4.36), and age ≥ 70 years (AOR: 3.96; 95% CI: 2.21, 7.10) were significantly associated with low vision. *Conclusion and Recommendation*. The prevalence of low vision found in this study was high as compared with the national and global magnitude. Older age, being female, previous history of cataract surgery, and no formal education were independently and significantly associated with low vision. Cataract and uncorrected refractive errors were identified as the main causes of low vision. Therefore, it requires a plan to provide an eye care education to the community, increasing the quality of cataract surgery and refractive service for the community in the catchment area.

## 1. Introduction

Low vision is defined as a presenting visual acuity (VA) of less than 6/18 but equal to or better than 3/60 in the better eye. It includes both moderate and severe visual impairments [1]. According to the Lancet Global Health Estimate in 2015, about 217 million people had low vision globally, of whom 118.9 and 172.3 million people with low vision were females, and their age was greater than 50 years and above, respectively [2].

On the other hand, a systematic review showed that the prevalence of low vision was 4.0% in sub-Saharan Africa. It is also reported that the number of people with low vision was varied in the subregion of Africa which ranges from the highest in West Africa (7.2 million) to lowest in Central Africa (1.4 million) [3]. Ethiopia is believed to have one of the world's highest rates of low vision (3.7%), of which more than 91.2% are either treatable or preventable causes [4]. Low vision can cause physical, economic, and psychological impact which leads to a reduced quality of life. In another way, when a person with low vision is not able to perform job-related functions at the workplace; loss of income and dependency occur [5].

Population-based studies conducted in Asian countries reported that the prevalence of low vision ranges from 3.09% to 22.1% [6–12]. On the other hand, institution-based research studies done in Africa showed that the prevalence of low vision extends from 17.1% to 28.2% [13, 14].

In Ethiopia, a national survey of low vision and blindness in 2005/6 showed that the prevalence of low vision was 3.7% and that of blindness was 1.6% [4]. Besides, a community-based study in the Gurage Zone, Southern Ethiopia, reported that the prevalence of low vision was 12.1% [15]. However, a hospital-based study in Addis Abeba, Central Ethiopia, showed that the prevalence of low vision was 10.3% [16].

Low vision is a priority agenda for Vision 2020, and it is also a public health problem in Ethiopia. As evidenced by outpatient registration, low vision is more common among the adult population attending the University of Gondar tertiary eye care and training center. The main objective of this study was to assess the proportion and associated factors of low vision among the adult population attending the University of Gondar tertiary eye care and training center. Also, this study will provide evidence to plan a way of improving an eye care service for the community in the catchment area of the hospital. Additionally, it helps to facilitate resource mobilization in the eye care system and low vision service.

## 2. Method and Materials

### 2.1. Study Design, Period, and Area

A hospital-based cross-sectional study was employed at the University of Gondar tertiary eye care and training center in Gondar Town from April 18 to May 16, 2019. Gondar Town is located 738 kilometers away from Addis Ababa, the capital city of Ethiopia. The University of Gondar tertiary eye care and training center is the only tertiary eye care center in the Amhara region providing comprehensive eye care service for the Northwest region of the country which comprises a total population of about 5 million. It has senior ophthalmologists, cataract surgeons, optometrists, ophthalmic nurses, and other assistant professionals with advanced ophthalmic equipment. As evidenced by the registration logbook of the center, the University of Gondar tertiary eye care and training center gives different services for about 31,200 patients per year in both outpatient and inpatient departments. It provides medical therapy, surgical services, laser services, and refraction with optical correction. There is also a newly established low vision clinic (since September 2018) in the center that provides low vision examination and low vision aids even though there is a scarcity of aids.

### 2.2. Study Population and Eligibility Criteria

The study participants were patients attending at the University of Gondar tertiary eye care and training center for an eye examination during the time of data collection. All patients, 18 years of age and above, were eligible to participate in the study.

### 2.3. Ethical Approval

Ethical clearance was obtained from the Institutional Ethical Review Board at the University of Gondar, College of Medicine and Health Sciences, School of Medicine. Oral informed consent was obtained from all participants after explaining the purpose of the study. Participants were informed about their right to withdraw from the study at any time during the interview and examination. The selected study subjects were not receiving any incentive or no risk would be imposed. The privacy and confidentiality of the study participants were secured by avoiding personal identifiers from data collection tools, and the data were locked with a password. The study participants with low vision were immediately linked to particular specialty and refraction clinics.

### 2.4. Sample Size Determination and Sampling Procedure

The sample size was determined based on the assumption that the expected proportion of low vision was 10.3% taken from a similar study in Addis Ababa [16], with 95% confidence level and the maximum allowable error of 2%. Then, the sample size was modified for a finite population with a reduction formula as the average number of patients come to the center per month was about 2600. Finally, 10% was considered for the nonresponse rate. A systematic random sampling technique with an interval of 4 was employed to select the study subjects. A single number was taken from 1 to 4 with a lottery method to determine the first subject and continue with every 4 intervals. The interval was calculated by dividing the expected number of patients come to the center per month to the total sample size (*N* = 2600; *n* = 727; *K* = (2600/727) = 4).

### 2.5. Operational Definition

The low vision was defined according to the World Health Organization's revised definition of visual impartment; it included both moderate and severe visual impairment [1].

Moderate visual impairment was defined as a presenting visual acuity of less than 6/18 and better than or equal to 6/60 in the better eye, whereas severe visual impairment was defined as a presenting visual acuity of <6/60 and better than or equal to 3/60 in the better eye.

The low vision was defined as a presenting visual acuity (VA) of less than 6/18 but better than or equal to 3/60 in the better eye.

### 2.6. Data Collection Procedures (Personnel and Instrument)

Five trained optometrists have participated in data collection. The training was given for data collectors on how to collect the data by the principal investigator. During the data collection, the reliability between raters was checked which gave a kappa score of 0.82. The data were collected using a structured questionnaire combined with a physical eye examination. The questionnaire includes questions covering the sociodemographic and economic data, behavioral factors, past ocular and medical history. In ophthalmic eye examination, distance visual acuity was measured using the distance Snellen acuity chart with tumbling E optotypes at a distance of 6 meters in good illumination. Pinhole vision was taken for individuals whose distance visual acuity was less than 6/9 to exclude whether the reduction of visual acuity is due to refractive error or not. If pinhole visual acuity was better than the baseline visual acuity, refractive error was considered as the cause of low vision.

However, a refractive error was considered as the cause of low vision after full eye examination was done to exclude any ocular condition that got improved through a pinhole.

Both anterior and posterior segment eye examination was done to identify ocular conditions that cause low vision. The anterior segment eye examination was done using a slit-lamp biomicroscope (SLB). However, the posterior segment (retina and macular) eye examination was performed with the use of 90 Diopter Volk lens and slit-lamp biomicroscope through a dilated pupil.

### 2.7. Data Quality Assurance

Data quality was controlled by using a pretested structured questionnaire. Training was given to data collectors (optometrists) and supervision was also made by a senior optometrist during data collection. The completeness of the data was checked onsite during the data collection and data entry period by the principal investigator.

### 2.8. Data Processing and Analysis

The collected data were coded and entered into Epi Info version 7 and then exported to SPSS version 20 for analysis. Descriptive statistics such as frequency distribution and measure of central tendency were used to summarize the descriptive part of the study. Bivariable logistic regression followed by a multivariable binary logistic regression model was fitted to determine the association between dependent and independent variables. The enter method was used to select a significant variable, and the fitness of the model was assessed using the Homer–Lemeshow test. The odds ratio with a 95% confidence interval was computed to find out the strength of the association between the independent and dependent variables. A variable with a *P* value of less than 0.05 was considered as statistically significant.

## 3. Results

### 3.1. Sociodemographic Characteristics of Study Participants

A total of 715 study participants participated in the study with a response rate of 98.6%. The mean age of study participants was 49.39 ± 19.93 years (range from 18 to 90 years). Among study participants, 421 (58.9%) were males and 370 (51.7%) were living in rural areas. Most of the study participants were currently married (562 (73.6%)). Of the total participants, 441 (61.7%) had no formal education (Table 1).

### 3.2. Past Ocular, Medical, and Behavioral Characteristics of Study Participants

Among the study participants, 70 (9.79%) were having a history of cataract surgery and 28 (3.92%) had a history of hypertension. Out of the total participants, only 36 (5.03%) of participants used eye-protective sunglasses (Table 2).

### 3.3. Magnitude of Low Vision

The prevalence of low vision in this study was 35.7% (95% CI: 32.3, 39.3). The main causes of low vision in this study were cataract (115 (44.1%)) and refractive error (45 (17.65%)) (Table 3).

### 3.4. Factors Associated with Low Vision

By applying bivariable analysis, low vision was positively associated with age, sex, marital status, residence, educational level, occupation, monthly income, and previous history of cataract surgery. However, in a multivariable logistic regression, older age, being female, no formal education, and previous history of cataract surgery were remained significantly associated with low vision. Regarding the educational level of study participants, those who have no formal education were 2.24 times more likely to have low vision than those who have formal education (AOR = 2.24; 95% CI: 1.25, 4.02).

Participants who had history of cataract surgery were 2.58 times more likely to have low vision as compared with their counter parts (AOR = 2.58; 95% CI: 1.53, 4.36). In addition, being female was 1.58 times more likely to have low vision than male (AOR = 1.58; 95% CI: 1.10, 2.28). Study participants whose age greater than or equal to 70 years old were 3.96 times more likely to have low vision as compared with those whose age less than or equal to 40 years old (AOR = 3.96; 95% CI: 2.21, 7.10) (Table 4).

## 4. Discussion

The burden of low vision in Ethiopia is a public health problem and known to have an impact on socioeconomic value and quality of life in the community. This study was aimed to determine the proportion and associated factors of low vision so that stakeholders will have evidence to plan and implement a strategy for improving an eye care service.

In this study, the prevalence of low vision was 35.7% (95% CI: 32.3, 39.3). This result is higher than other previous hospital-based studies done in Addis Abeba, Ethiopia (10.3%) [16], Ghana (28.2%) [14], and South Africa (17.1%) [13]. This difference might result from variation in sampling technique in which the study in South Africa used nonprobability sampling and the data collection techniques, and data source in the Ethiopian and the Ghanian studies were medical records as well as it was done with a large sample size which may contribute for the discrepancy beteween the two studies. Besides, the variation in a socioeconomic characteristic of the study population could also contribute to this difference. On the other hand, the result of this study is higher than studies done in the Gurage Zone of Ethiopia (12.1%) [15] and a national survey of Ethiopia (3.7%) [4], Afghanistan (13.9%) [9], Sri Lankan (19.6%) [10], Indian states (9.2–22.1%) [8, 11, 12], Malaysia (7%) [7], and Iran (3.09%) [6]. This difference might be due to the variation in sampling design and sample size in which those studies were done at the community level with a larger sample size. Additionally, the variation in accessibility and availability of eye care service and socioeconomic status of the study population play a role in this difference [11, 17].

In this study, 40.82% of females had lived with low vision, and being female was 1.58 times more likely to have low vision than males. This finding is in line with studies conducted in the Gurage Zone of Ethiopia [15], Nepal [18], Indian states [17, 19], Saudi Arabia [20], and China [21, 22]. The possible reasons for this variation could be that females have had longer life expectancy than males, which in turn leads to developing eye conditions that could be increasing low vision [23]. Also, this might be happening because females could have unequal access for eye care services in some rural areas and the social stigma of wearing spectacles [18], whereas studies were done in Afghanistan, Sri Lankan, North India, and Iran reported that gender was not associated with low vision [6, 9–11].

Regarding the education level of a study participant, those who have no formal education were 2.24 times more likely to have low vision than those having a formal education. Similar findings were reported from studies conducted in Nepal [18], in different states of India [11, 17, 19], and China [24]. The possible reason is that noneducated individuals had low detailed visual demands to performed daily activities that may not enforce to seek the treatment. Also, poor awareness about the problem as well as service and fear for eye care intervention might be contributed to this finding [25].

Previous history of cataract surgery was found to be another determinant factor where those who had a history of cataract surgery were 2.58 times more likely to have low vision than their counterparts. However, this finding is new and unproven in previous studies and against the fact, for example, having surgery for pure cataract could lead to a better outcome rather than low vision. Despite this, the possible reason could be a failure to detect preexisting eye conditions (such as age-related macular degeneration (ARMD), glaucoma, and diabetic retinopathy). And also, postsurgical complications such as posterior capsule opacification (PCO) and postoperative large uncorrected refractive error could worsen the preexisting vision and lead to low vision [26, 27].

Older age was also another important factor in which age above 60 years old was more than 3 times at risk to develop a low vision. This finding is supported by studies done in the Gurage Zone of Ethiopia [15], Nepal [18], Sri Lanka [10], and the state of India [11, 17, 19]. The possible reason might be due to the common causes of low vision such as cataract, glaucoma, and age-related macular degeneration are age-related eye diseases [13, 14]. However, the study done in Afghanistan showed that age was not associated with low vision [9]. This difference might be due to a variation in age categorization.

As a limitation of the study, there is a scarcity of hospital-based studies in Ethiopia and other parts of Africa which makes the comparison and discussion of the findings with population-based studies. Additionally, the definition of low vision was made based on the presenting visual acuity which brings difficulty in estimating the proportion of low vision individuals who really need optical low vision aids at the time of the study.

## 5. Conclusion

The prevalence of low vision found in this study was high as compared with the global and national prevalence of low vision. Older age, being female, previous of history cataract surgery, and no formal education were independently and significantly associated with low vision. Cataract and uncorrected refractive errors were the main causes of low vision. Therefore, it requires a plan to provide an eye care education to the community, increasing quality cataract surgery and refractive services. Additionally, researchers shall conduct further studies which clearly shows the need of low vision aids by considering the service provision aspect of low vision.

## Figures and Tables

**Table 1:** Sociodemographic and economic characteristics of study participants, the University of Gondar tertiary eye care and training center, Northwest Ethiopia, 2019 (*n* = 715).

Variables	Frequency	Percent (%)
Age (years)		
≤40	272	38
40–49	63	8.8
50–59	95	13.3
60–69	123	17.2
≥70	162	22.7

Sex		
Male	421	58.9
Female	294	41.1

Residence		
Rural	370	51.7
Urban	345	48.3

Marital status (currently)		
Single	189	26.4
Married	526	73.6

Education		
No formal education	441	61.7
Formal education	274	38.3

Occupation		
Farmer	259	36.2
Housewife	135	18.9
Retired	42	5.9
Student	87	12.2
Government employer	118	16.5
Others	74	10.3

Monthly income (Ethiopian birr)		
≤1000	261	36.5
1001–1500	152	22
1501–2500	128	17.9
>2500	169	23.6

Education categories, no formal education (can read and write or not) and formal education (primary school (1–8th), high school (8–12th), and college/university (diploma, degree, and above)).

**Table 2:** Past ocular, medical, and behavioral characteristics of study participants, the University of Gondar tertiary eye care and training center, Northwest Ethiopia, 2019 (*n* = 715).

Variables	Low vision		Frequency	Percent
	Yes	No		
History of cataract surgery				
Yes	11	59	70	9.79
No	244	401	645	90.2

History of any nonsurgical ocular injury				
Yes	23	33	56	7.83
No	232	427	639	92.17

History of diabetes mellitus				
Yes	8	18	26	3.64
No	247	442	689	96.36

History of hypertension				
Yes	16	12	28	3.92
No	239	448	687	96.08

Cigarette smoking				
Yes	5	6	11	1.54
No	250	454	704	98.45

Using eye-protective sunglass				
Yes	9	27	36	5.03
No	246	433	679	94.97

**Table 3:** Cause of low vision at the University of Gondar tertiary eye care and training center, Northwest Ethiopia, 2019 (*n* = 715).

Common ocular disorder	Frequency (%)	Low vision	
		Yes	No
Cataract	240 (33.57)	115	125
Refractive error	90 (12.59)	45	45
Glaucoma	70 (9.79)	30	40
Corneal opacity	60 (8.39)	25	35
Pseudophakia	70 (9.79)	11	59
Age-related macular degeneration	26 (3.64)	8	18
Diabetic retinopathy	20 (2.80)	6	14
Ocular surface diseases	86 (12.02)	5	81
Others	53 (7.41)	10	43
Total	715 (100%)	255	460

Others: uveitis, retinal detachment, hypertensive retinopathy, optic neuritis, phthisis bulbi, macular hole, and optic atrophy. Ocular surface diseases: blepharitis, conjunctivitis, and pterygium.

**Table 4:** Factors associated with low vision among adult patients attending the University of Gondar tertiary eye care and training center, Northwest Ethiopia, 2019 (*n* = 715).

Variables	Low vision			AOR (95% CI)
	Yes	No	COR (95% CI)	
Age (years)				
≤40	39	233	1.00	1.00
40–49	17	46	2.21 (1.15, 4.24)	1.35 (0.67, 2.76)^*∗*^
50–59	41	54	4.54 (2.67, 7.70)	2.27 (1.22, 4.22)^*∗∗∗*^
60–69	65	58	6.70 (4.10, 10.93)	3.25 (1.80, 5.87)^*∗∗∗*^
≥70	93	39	8.05 (5.08, 12.76)	3.96 (2.21, 7.10)^*∗∗∗*^

Sex				
Male	135	286	1.00	1.00
Female	120	174	1.46 (1.07, 1.99)	1.58 (1.10, 2.28)^*∗∗*^

Residence				
Rural	167	203	2.40 (1.75, 3.30)	0.96 (0.64, 1.45)
Urban	88	257	1.00	1.00

Marital status (currently)				
Single	42	147	0.42 (0.29, 0.62)	0.87 (0.54, 1.40)
Married	213	313	1.00	1.00

Education				
No formal education	215	226	5.57 (3.79, 8.17)	2.24 (1.25, 4.02)^*∗∗*^
Formal education	40	234	1.00	1.00

Occupation				
Farmer	118	141	5.34 (2.98, 9.54)	1.31 (0.52, 3.30)
Housewife	69	66	6.67 (3.57, 12.46)	1.81 (0.70, 4.73)
Retired	24	18	8.50 (3.79, 19.05)	2.29 (0.80, 6.52)
Student	10	17	0.83 (0.36, 193)	1.40 (0.50, 4.00)
Others	18	56	2.05 (0.97, 4.33)	1.04 (0.43, 2.57)
Government employer	16	102	1.00	1.00

Monthly income (Ethiopian birr)				
≤1000	106	155	2.93 (1.85, 4.63)	0.99 (0.55, 1.76)
1001–1500	65	92	3.03 (1.84, 4.99)	0.97 (0.53, 1.77)
1501–2500	52	76	2.93 (1.74, 4.94)	1.05 (0.57, 1.95)
>2500	32	137	1.00	1.00

History of cataract surgery				
Yes	55	28	4.24 (2.61, 6.89)	2.58 (1.53, 4.36)^*∗∗*^
No	197	429	1.00	1.00

^*∗*^Nonsignificant, ^*∗∗*^*P* < 0.01, and ^*∗∗∗*^*P* < 0.001. CI, confidence interval; COR, crude odds ratio; AOR, adjusted odds ratio.

## Data Availability

All the necessary data are included in the manuscript, and if needed, the supporting data are availed.
